# Universal CD7-targeted CAR T-cell therapy in T-ALL: a promising bridge to transplant, but not without caveats—a critical reappraisal

**DOI:** 10.3389/fonc.2026.1856798

**Published:** 2026-06-11

**Authors:** Qinghua Ke, Shiqiong Zhou

**Affiliations:** Department of Chemoradiotherapy, The First People’s Hospital of Jingzhou, Jingzhou, Hubei, China

**Keywords:** allogeneic stem cell transplantation, antigen escape, base editing, T-cell acute lymphoblastic leukemia, T-cell aplasia, therapeutic paradox, universal CAR-T cells

## 1 Introduction

The phase 1 study by Chiesa and colleagues is an important proof of concept, demonstrating that allogeneic, base-edited anti-CD7 CAR T-cells (BE-CAR7) can induce morphologic remission in heavily pretreated patients with relapsed/refractory T-ALL (1). The fact that most of these patients could then proceed to allogeneic HSCT is not trivial for a disease with such a dismal post-relapse prognosis. However, as a clinician, I read these results with cautious skepticism rather than uncritical optimism. Based on only 11 patients and limited follow-up, the study answers some questions but raises several more pressing ones. The central issue is no longer whether BE-CAR7 can work—it clearly can—but rather whether the magnitude of benefit justifies the extraordinary complexity, cost, and toxicity. Is this a genuine breakthrough, or an elaborate, resource-intensive bridge that still leaves patients vulnerable? This commentary argues that it is currently the latter and that the field must confront the therapeutic paradox head-on: using a highly sophisticated gene-edited product primarily as a preparatory step for transplant, rather than as a definitive cure.

## 2 Core contributions: real but narrow

The study’s main achievement is demonstrating that a “one-size-fits-all” triple-edited cell product (TCRαβ^-^/CD52^-^/CD7^-^) is technically feasible and can induce deep, MRD-negative remissions (1). The 82% rate of successful bridging to HSCT in this heavily pretreated population is clinically respectable. As the authors correctly note, this is a bridging therapy, not a definitive cure, but it creates a window for a potentially curative transplant.

That said, we should not confuse feasibility with superiority. The “off-the-shelf” nature is indeed an advantage for rapidly progressive T-ALL, but whether this model is scalable to real-world practice—where HLA diversity, manufacturing reproducibility, and cost constraints come into play—remains entirely open. We also need to acknowledge that the study does not compare BE-CAR7 to anything. Without a control arm, we simply do not know whether these patients would have done equally well with a modern salvage regimen (e.g., nelarabine-based) followed by transplant.

## 3 Unresolved clinical and biological challenges

### 3.1 The therapeutic paradox: bridge or expensive detour?

The most underdiscussed issue in the original study—and in current discourse—is the therapeutic paradox. We are deploying a multiplex base-edited allogeneic product that requires intensive alemtuzumab-based lymphodepletion, close monitoring for viral reactivation, and then still mandates a full HSCT. The patient endures prolonged T-cell aplasia, infection risk, and the cumulative toxicity of two sequential cellular therapies—yet the BE-CAR7 itself is not intended to be curative. From a clinical standpoint, this raises an uncomfortable question: if the patient is eventually going to receive a transplant anyway, what exactly does BE-CAR7 add over a less toxic, less expensive salvage chemotherapy that also achieves MRD negativity? The study does not answer this, and until a randomized trial is done, we should resist the temptation to assume that “newer” means “better”.

### 3.2 Overinterpreting small numbers

The n=11 cohort is the elephant in the room (1). We cannot speak of durable cures when follow-up post-HSCT is only 3–36 months. The study was designed to assess safety and bridging potential, not long-term CAR-T-attributable cure. The real test will be how many of these bridged patients remain in remission and off immunosuppression years later. Until then, statements about “transformative potential” must be heavily qualified—and preferably avoided.

### 3.3 Antigen escape: a different beast from CD19

The two patients with CD7-negative relapse highlight a key vulnerability (1). What caught my attention is that the authors found no escape mutations in the CD7 exons, which points away from the selection-driven clonal evolution we often see with CD19 CAR-T cells (2). Instead, non-genetic mechanisms like epigenetic silencing or trogocytosis are plausible, but we need mechanistic data (3). From a clinical perspective, this is worrying: if CD7 loss is reversible or preexisting in minor subclones, then even dual targeting (e.g., CD7/CD5) might not fully solve the problem. This mechanism deserves its own dedicated study, including single-cell sequencing of pre- and post-relapse samples.

### 3.4 Prolonged T-cell aplasia: the price of pan-T-cell targeting

The original commentary touched on viral reactivations but did not go far enough. We are targeting CD7, an antigen expressed on virtually all normal T cells. So, it should not be surprising that all patients had persistent cytopenias through day 28, and viral reactivations were detected universally. The question is not whether this happens, but how we manage it in routine practice—and whether the current alemtuzumab-containing lymphodepletion regimen is simply too toxic (4). One could argue that the benefit of protecting BE-CAR7 from host rejection must be weighed against the real risk of life-threatening opportunistic infections. The study reported serious post-transplant viral complications in 3 of 11 patients—that is a signal, not noise. Future trials should carefully explore whether reduced-intensity lymphodepletion (perhaps with a different serotherapy backbone) can preserve CAR-T expansion without leaving patients defenseless for months.

### 3.5 Base editing: elegant but not proven safer

The manuscript conveys a somewhat optimistic view of base editing without adequately discussing important concerns. While base editors reduce double-strand breaks compared to CRISPR nucleases (5), they are not without risks—including off-target single-base mutations, guide RNA-dependent off-target editing, and potential for unanticipated transcriptome-wide effects (6). Long-term safety data in humans are virtually non-existent (7). We also lack clarity on whether the triple-edited BE-CAR7 product might have unexpected immunogenicity or delayed genotoxicity. Until we have larger trials with long-term integration site analysis and secondary malignancy monitoring, we should treat base editing as an unproven tool in the clinical setting, not a clear improvement over nuclease-based approaches.

## 4 Comparative perspective: more questions than answers

Several allogeneic CD7-directed approaches are in development. **Table 1** provides a comparison, but with an emphasis on what we still do not know—particularly regarding persistence, off-target risks, and comparative efficacy.

**Table 1:** Comparative overview of allogeneic CD7-directed CAR-T platforms (adapted and expanded).

Feature	BE-CAR7 (Chiesa et al.)	CRISPR-Cas9-edited universal CD7 CAR	Donor-derived CD7 CAR (TCR-depleted)	Protein-blocker-based fratricide inhibition
Gene-editing tool	Cytidine base editor (CBE)	CRISPR-Cas9 nuclease	None (viral vectors only)	None (recombinant protein blockers)
Key modifications	TCRαβ^-^/CD52^-^/CD7^-^ functional disruption	TCRαβ^-^/CD7^-^ knockout	Typically TCRαβ-depleted or edited	Transient surface CD7 blockade
Off-the-shelf potential	High	High	Low (donor-dependent)	Variable
Fratricide prevention	Genetic disruption	Genetic knockout	Often incomplete	Pharmacological inhibition
Clinical stage	Phase 1	Early phase 1/2	Phase 1/2	Preclinical/early clinical
Reported MRD-negative remission rate	82% (9/11) bridging to HSCT	Variable, <50%–80%	70%–90% in small series	Not yet reported
Known persistence	Unknown beyond lymphodepletion	Variable	Weeks to months	Short-lived by design
GVHD risk	Very low (TCRαβ-edited)	Very low	Low to moderate if TCR-depleted	Low
Manufacturing complexity	Very high (multiplex base editing)	High	Moderate (donor leukapheresis)	Low (protein production)
Primary advantages	Precise editing, reduced DSB risk, triple disruption	Established platform, efficient knockout	Simpler manufacturing, no editing	Avoids genetic manipulation, transient effect
Key limitations	Scalability, long-term editing safety, prolonged aplasia	Off-target effects, chromosomal rearrangements	GvHD risk, persistence issues	Transient effect, unknown *in vivo* efficacy

As **Table 1** shows, each platform has trade-offs. BE-CAR7’s use of base editing is elegant (1), but we have far less long-term safety data on base editors than on nucleases (6). The triple disruption addresses multiple barriers simultaneously, but at the cost of profound and prolonged immunosuppression. Donor-derived CD7 CAR platforms have shown promising early results (8–10). No platform has shown clear superiority, and none has been tested head-to-head.

## 5 Real-world barriers and health-economic realities

### 5.1 Is a transplant always necessary?

The current commentary, like the original study, frames HSCT as the mandatory endpoint. However, we should at least ask the question: could a future generation of universal CAR-T cells be durable enough to spare patients the toxicity of a transplant? For that to happen, we would need CAR-T cells that persist long-term without causing GVHD and without continuous T-cell aplasia. That is a tall order, but not impossible. It is worth noting that in the study by Hu et al. (11), sequential CD7 CAR-T therapy and HSCT without GVHD prophylaxis was feasible, but that still required transplant. For now, BE-CAR7 is a bridge, but the field should keep an eye on transplant-sparing strategies.

### 5.2 Health-economic realities

The combined cost of a multiplex-edited allogeneic CAR-T product, intensive lymphodepletion, management of viral reactivations, and then a full HSCT is staggering. From a health-system perspective, we need cost-effectiveness analyses. Is this sequence truly superior to modern salvage chemotherapy followed by transplant? We do not know, because no head-to-head trial exists. Moreover, in resource-limited settings, this regimen is simply not feasible—it requires a highly specialized center with expertise in cellular therapy, transplant, and infectious disease management. This will exacerbate existing inequities in cancer care.

Beyond biology, we must confront the practical reality: the combined cost of a multiplex-edited allogeneic CAR-T product, intensive lymphodepletion, management of viral reactivations, and then a full HSCT is staggering. From a health-system perspective, we need cost-effectiveness analyses. Is this sequence truly superior to modern salvage chemotherapy followed by transplant? We do not know, because no head-to-head trial exists. In resource-limited settings—which include many public hospitals even in high-income countries—this regimen is simply not feasible. It requires a highly specialized center with expertise in cellular therapy, transplant, and infectious disease management. This will exacerbate existing inequities in cancer care. As editors and clinicians, we have a responsibility to ask not only “does it work?” but also “for whom, at what cost, and compared to what?”

## 6 Future directions: from hype to hypothesis

This study is a starting line, not a finish line. Here is what I would want to see next:

1. Optimize, don’t just intensify. We need a phase 2 trial comparing the current alemtuzumab-based lymphodepletion to a reduced-intensity regimen. The endpoint should be not just CAR-T expansion but also infection-free survival at day 100 post-HSCT.
2. Mechanistic studies of CD7 loss. Before investing heavily in dual-targeted CARs, we need to understand whether CD7 loss is genetic (even if not in exons), epigenetic, or due to trogocytosis. Single-cell sequencing of pre- and post-relapse samples is essential.
3. Randomized comparisons. A randomized phase 2 trial comparing BE-CAR7 → HSCT versus novel salvage chemotherapy (e.g., nelarabine-based) → HSCT is the only way to prove superiority. Until then, this remains a promising but unproven strategy.
4. Long-term safety monitoring for gene editing. Mandatory long-term follow-up for off-target mutations, vector integration sites, and secondary malignancies should be part of any protocol involving base-edited products. The field has learned painful lessons from earlier gene therapy trials; we should not repeat them.

## 7 Conclusions

Chiesa and colleagues have done a commendable job showing that universal BE-CAR7 cells can debulk refractory T-ALL and enable transplant. However, as a clinician, I see a therapy that still carries substantial risks of prolonged T-cell aplasia, viral reactivation, antigen escape, and uncertain long-term safety of base editing. It is a promising bridge, but a bridge that remains poorly characterized, expensive, and potentially replaceable by simpler strategies. The goal must now shift from simply getting patients to transplant to asking whether this particular bridge is necessary, cost-effective, or superior to existing options. That will require larger, randomized trials, mechanistic studies, and a more critical, hypothesis-driven approach—not uncritical enthusiasm.
